# The Use of Vasodilator Therapy in Fontan Patients: A Single-Centre Experience

**DOI:** 10.3390/children12060751

**Published:** 2025-06-10

**Authors:** Alessia Faccini, Martina Avesani, Roberta Biffanti, Elettra Pomiato, Domenico Sirico, Alice Pozza, Alessia Cerutti, Elena Reffo, Biagio Castaldi, Giovanni Di Salvo

**Affiliations:** Division of Paediatric Cardiology, Department of Women’s and Children’s Health, University Hospital Padua, 35128 Padua, Italy; alessiafaccini@hotmail.com (A.F.);

**Keywords:** Fontan circulation, pulmonary arterial hypertension, pulmonary vasodilator therapy

## Abstract

Background: The aim of this study was to describe our centre experience in the use of pulmonary vasodilator therapy in Fontan patients. Methods: We retrospectively enrolled patients that underwent Fontan operation between 2000 and 2024, reporting demographic and operative data and noting complications and the use of pulmonary vasodilators. Results: A total of 117 patients were followed for a median time of 150 months (90–207). In total, 36.7% were female, and the median age during the intervention was 50 months (37–64), and 53% had a single left ventricle physiology. In 20 of these 117 patients (17.1%), at least one pulmonary vasodilator drug was used during their life for the following reasons: 6 elevated pressures in the circuit, 3 low oxygen saturation, 2 plastic bronchitis, 2 pleural effusion, 1 chylothorax, 1 persistent pericardial effusion, 1 haemoptysis, 1 protein losing enteropathy, 1 poor exercise tolerance, 1 pulmonary arterial hypertension present since birth and 1 diastolic dysfunction. They had a significantly higher prevalence of single right ventricle physiology (65% vs. 37%, *p* = 0.03), pulmonary hypertension (60% vs. 0, *p* = 0.0001), plastic bronchitis (10% vs. 0, *p* = 0.03) and declivous oedema in the follow-up period (10% vs. 0, *p* = 0.03), with a higher assumption of warfarin (35% vs. 6.2%, *p* = 0.001). Conclusions: We found that in the absence of a standardise protocol, we usually use pulmonary vasodilator therapy in Fontan patients, as it is guided by clinical aspects and hemodynamic conditions, which lead us to start and stop this therapy.

## 1. Introduction

Described for the first time in 1971 [1,2], the Fontan procedure is the last surgical step in the palliation of patients with univentricular physiologies. Typically performed following the Glenn operation, the Fontan procedure redirects the entire systemic venous return directly to the pulmonary arteries, thereby avoiding mixing between oxygenated and deoxygenated blood. Over time, the technique evolved, with the extracardiac conduit (a prosthetic graft connecting the inferior vena cava to the pulmonary arteries, bypassing the right atrium) and the lateral tunnel (an intra-atrial baffle constructed within the right atrium to channel inferior vena cava flow to the pulmonary arteries) now being the most commonly employed approaches [3,4]. Additionally, in patients considered at higher risk, a fenestration—either in the atrial baffle or the conduit—is often created to preserve cardiac output and reduce atrial pressure [5].

In Fontan circulation, where the systemic venous return is passively directed to the pulmonary arteries without a subpulmonary pump, blood flow through the lungs relies solely on the residual postcapillary energy. The inherent pulmonary vascular impedance hinders venous return, resulting in upstream congestion and limited downstream flow [6].

Therefore, the resistance of the capillary bed is the critical determinant of output. Indeed, low pulmonary vascular resistance is necessary to sustain adequate pulmonary blood flow at low central venous pressure. However, at the time of the Fontan operation, the pulmonary vasculature is already partially compromised. This deterioration arises either from reduced pulmonary blood flow, leading to a decreased pulmonary vascular cross-sectional area, or from increased pulmonary flow and pressure, which induce pathological vascular remodelling [7]. Moreover, the reduction in the expression of endothelial nitric oxide synthetase and the enhancement of vascular endothelial growth factor and endothelin production seem to contribute to the development of pulmonary hypertensive vascular disease [8], which has been recently defined by The European Paediatric Pulmonary Vascular Disease Network (EPPVDN) and the American Heart Association as a transpulmonary gradient of >6 mmHg and a PVR index of >3 WU × m^2^ [9,10].

In this hemodynamic condition, the use of pulmonary vasodilator drugs, such as endothelin receptor antagonists and phosphodiesterase-5 inhibitors, may be helpful. While these therapies are given with wide consensus in symptomatic patients with overt Fontan failure, conflicting results and insufficient evidence do not allow for their routinely recommendation across this population [11,12,13,14,15,16,17]. Thus, the administration of these medications is chiefly based on the clinical experience of individual centres, rather than on established standardised protocols. In patients with pulmonary arterial hypertension (PAH) without congenital heart disease, current guidelines recommend initiating pulmonary vasodilator therapy based on specific haemodynamic data obtained via cardiac catheterization, typically advocating for combination therapy as the initial approach. In contrast, for patients with congenital heart disease, particularly those with univentricular physiology, standardised treatment protocols are lacking, and current practice favours starting with monotherapy rather than combination therapy [18].

Therefore, the aim of this study was to analyse the experience of pulmonary vasodilators administration in Fontan patients at our centre in order to understand factors influencing both the initiation and discontinuation of this therapy.

## 2. Materials and Methods

In this retrospective study, which was conducted between March and July 2024, we reviewed clinical data of patients who underwent Fontan completion between January 2000 and March 2024. For each patient, we recorded demographic parameters, age at intervention, baseline diagnosis, and the type of Fontan procedure performed. Additionally, we documented perioperative complications, early and late mortality, clinical issues arising during follow-up, the need for additional procedures, and the antithrombotic therapy administered. Finally, we collected data from cardiac catheterization pre- and post-Fontan completion and analysed the type of pulmonary antihypertensive drug used, the reason for initiating therapy, and its duration.

## 3. Statistics

Given the small sample size, a normal distribution was not assumed. Therefore, continuous values are presented as the median and interquartile range (IQR). The non-parametric Mann–Whitney U test and the Wilcoxon signed-rank test were used to compare the median values between the independent and dependent groups, respectively. Categorical variables were analysed through Fisher’s exact test. A *p* < 0.05 was considered statistically significant.

## 4. Results

From January 2000 until March 2024, 133 patients underwent Fontan completion.

For a total of sixteen patients, comprehensive data could not be retrieved from our database: death occurred in 9 patients (6.8%; five died in the immediate post-operative period, while 4 died some years after the intervention), 4 (3%) patients underwent heart transplantation and were lost at follow-up, and 3 (2.2%) patients moved abroad.

Therefore, 117 patients, 36.7% of whom were females, were included in the analysis with a median follow up of 150 months (IQR of 90–207). Their median age at follow-up was 16 years (IQR of 13–20).

Forty-nine (41.9%) had a single right ventricle physiology, primarily due to hypoplastic left heart syndrome (HLHS). A total of 62 (53%) had a single left ventricle physiology, most commonly due to tricuspid atresia (TA), and 6 (5.1%) had an indetermined prevalent physiology.

The median age at intervention was 50 months (IQR of 37–64). One-hundred patients (85.5%) underwent extracardiac conduit surgery. The most common perioperative complication was pleural effusion (49.6%). Forty-nine patients (41.9%) underwent percutaneous procedures during the follow-up. Follow-up complications were dominated by arrhythmias, which was observed in 37 patients (31.6%). In terms of antithrombotic therapy, 82 patients (70%) were treated with acetylsalicylic acid. Demographic and anatomical data, along with details on the type of intervention, perioperative complications, clinical issues that emerged during the follow-up, the need for additional procedures, and the antithrombotic therapy administered, are shown in Table 1.

In 20 of 117 patients (17.1%), at least one pulmonary vasodilator drug was used. Detailed characteristics are provided in Table 2. Seven (35%) patients were female. Thirteen (65%) had a right ventricle physiology, and the median age during the Fontan intervention was 64 months (IQR of 55–71.25); all but two patients had an extracardiac conduit. In one case, pulmonary vasodilator therapy was started in the perinatal period; in two cases, it was started pre-Fontan (when the patients were, respectively, 1 years old and 2 years old); in two cases, it was started preoperatively, in three cases, it was during in the perioperative period. In 12 cases, it was started months or years post-intervention. Regarding medication use, 11 patients received bosentan, 6 were treated exclusively with sildenafil, and 3 were prescribed both drugs. Of these, two patients switched to sildenafil after discontinuing bosentan due to the recurrence of symptoms, while one patient took both medications concurrently. The primary indications for initiating pulmonary vasodilator therapy included elevated circuit pressures (6 patients), low oxygen saturation (3), plastic bronchitis (2), pleural effusion (2), chylothorax (1), persistent pericardial effusion (1), haemoptysis (1), protein-losing enteropathy (1), poor exercise tolerance (1), pulmonary arterial hypertension since birth (1) and diastolic dysfunction (1). Additionally, key haemodynamic parameters, such as mean pulmonary artery pressure, transpulmonary gradient, and indexed pulmonary vascular resistances, as well as anatomical findings prior to the initiation of vasodilator therapy, are presented. Twelve patients fell under the definition of pulmonary hypertension in Fontan patients during their lives. Pulmonary vasodilators were started without performing diagnostic cardiac catheterization in two patients, and in one case, the hemodynamic study was performed before starting bosentan but not before sildenafil. At the end of the follow-up, 13 of them (65%) were still on therapy (6 patients were on bosentan, 4 on sildenafil, 2 on sildenafil after bosentan discontinuation and 1 on both drugs). In seven (35%) patients (five taking bosentan and two taking sildenafil), pulmonary vasodilator therapy was discontinued following the resolution of the primary condition that led to its initiation: resolution of plural effusion (2 cases), improved plastic bronchitis following stent positioning in the stenotic left pulmonary artery (1), and normalised circuit pressures (4). In the remaining cases, pulmonary vasodilator drugs allowed coping with basal clinical conditions for several years with mild but not substantial improvement.

The baseline characteristics, surgical interventions, perioperative course, and follow-up data were compared between patients who received pulmonary vasodilators and those who did not (Table 3). While the two groups were comparable in terms of the adopted surgical approach, the treated group was older, had a higher prevalence of single right ventricle physiology (*p* = 0.03), a trend of an increased occurrence of arrhythmias in the perioperative period (*p* = 0.06), and a higher prevalence of plastic bronchitis (*p* = 0.03) and declivous oedema (*p* = 0.03) in the follow-up; and seemed to assume warfarin more often (*p* = 0.001). Non-treated patients had a longer follow-up period. As mentioned above, 12 patients in the treated group had a diagnosis of pulmonary hypertension during their lifetime, whereas no patients in the non-treated group had pulmonary hypertension (*p* = 0.0001).

## 5. Discussion

The key findings of this retrospective study are that pulmonary vasodilator drugs in patients with univentricular physiology are administered at our centre in a higher proportion of patients compared to the average reported in the literature. Additionally, the indications for initiating and discontinuing treatment as well as the timing vary and appear to follow a more personalised approach rather than a well-defined protocol.

### 5.1. Comparison with Literature Data

A recent UK survey found an overall usage rate of 4.9%, with only one centre reporting administration in 14% of patients [19], while in our centre 17.1% of patients received pulmonary vasodilator therapies. This could reflect not only the well-known lack of defined guidelines and protocols about the use of these drugs [13,14,15] but also the variable and sometimes unpredictable response of Fontan physiology to available therapeutic options [8]. In the above-mentioned survey, pulmonary vasodilators were not administered routinely in all Fontan patients but only in precise conditions such as non-respondent protein-losing enteropathy, untreatable fluid overload and frequent heart failure admissions. The findings of the present study are consistent with this data. By contrast, the population included in our study is younger and includes only patients treated with an extracardiac conduit or a lateral tunnel. Also, bosentan was administered slightly more frequently than sildenafil as the initial therapy, reflecting clinical preferences. The primary indication for initiating therapy in our cohort was elevated pulmonary pressure, with percentages comparable to those reported in the survey (30% vs. 36% in the UK study). However, poor exercise tolerance was less common in our population, and we observed one case of plastic bronchitis, which was not reported in the UK study. Additionally, our cohort showed a greater use of acetylsalicylic acid compared to anticoagulant therapy. Notably, in contrast to the survey findings, 35% of our patients discontinued pulmonary vasodilator therapy due to the resolution of the underlying condition. These differences suggest that pulmonary vasodilator therapy may be more effective in certain clinical contexts, underscoring the need for further research to establish more specific criteria for its use in this heterogeneous patient population.

In this regard, while the use of pulmonary vasodilator therapy in symptomatic patients with overt Fontan failure has become part of routine clinical practice [11,12,17], there is insufficient evidence to recommend its routine use across all Fontan patients.

### 5.2. Pathophysiological and Clinical Reasons to Use Pulmonary Vasodilators

In theory, pulmonary vasodilator therapy in Fontan patients should positively influence a circulation dependent on pulmonary vascular resistance, but the timing and indications to start it are still a matter of debate, and conflicting results exist about its beneficial effects on perioperative outcomes, symptoms and exercise tolerance [13,14,15]. Indeed, some studies in patients without Fontan failure suggest an improvement in exercise capacity [14], while others contradict these findings [13].

In addition, in Fontan physiologies, the circulatory issues are often more related to chronic volume depletion and altered loading conditions rather than a purely restrictive pulmonary vasculature [20].

However, in this study, 12 patients (60%) met the definition of pulmonary hypertension during their life [9,10]. This condition was the unique reason for initiating vasodilator therapy in only six cases. In the remaining six patients, it served as additional support for the clinical indication, since cardiac catheterization was performed after complications such as plastic bronchitis or persistent pleural and pericardial effusions developed. This suggests that a systematic haemodynamic study could lead to early diagnoses and subsequently treat pulmonary hypertension, possibly preventing further complications.

When comparing patients treated with vasodilator drugs with those not treated, the former underwent Fontan completion later than the latter. A higher prevalence of single right ventricle physiology was also noted in the treated group. This could indicate a potential pulmonary overflow before Glenn and Fontan that could eventually lead to pulmonary vasodilators usage [21]. In addition, patients who received vasodilator therapies showed a higher prevalence of complications such as plastic bronchitis and declivous oedemas and underwent more percutaneous interventions following Fontan completion. Finally, warfarin was more often administered to patients on pulmonary vasodilators. All these data suggest more unstable haemodynamic conditions and a higher tendency of complications in treated patients, confirming data already present in the literature [17].

## 6. Study Limitations and Future Perspectives

Due to its retrospective and monocentric nature, our study has several limitations. Firstly, it is based on a small sample size, focusing only on patients for whom data were available; therefore, caution is needed when interpreting the statistically significant data. Secondly, data on functional capacity are lacking; a prospective trial incorporating cardiopulmonary exercise testing or, if not feasible, alternative functional markers such as SaO2, the NYHA functional class or brain natriuretic peptide levels before and after the introduction of pulmonary vasodilators could offer additional insights into functional capacity. Thirdly, not all patients underwent cardiac catheterization prior to initiating therapy. Implementing a standardised pre-treatment protocol would be beneficial for obtaining more interpretable data and ensuring more precise selection of patients. Moreover, the follow-up period in our study is limited, and long-term outcomes require further investigation. Finally, multi-centre, large-scale studies should be conducted to obtain more consistent data and potentially establish standardised criteria for initiating and discontinuing pulmonary vasodilator therapy.

## 7. Conclusions

Pulmonary vasodilator therapy usage in Fontan patients is still a matter of debate in terms of timing and indications, with a global consensus in symptomatic patients with overt Fontan failure, but there is insufficient evidence to recommend its routine use across all populations. In our hospital, in the absence of a standardised protocol, pulmonary vasodilator therapy is typically used in Fontan patients based on clinical factors and hemodynamic conditions, which guide the decision to initiate and discontinue the therapy. The current literature does not provide universally recognised standardised protocols and demonstrates variability in the use of pulmonary vasodilator therapy in Fontan patients. This highlights the need for further research and greater efforts to deepen our understanding of this unique circulation, ultimately aiming to improve outcomes for these patients.

## Figures and Tables

**Table 1:** Demographic and anatomical characteristics and perioperative and follow-up data.

Female (n, %)	43 (36.7%)
Age at intervention, months (median, IQR)	50 (37–64)
Single right ventricle physiology (n, %)	49 (41.9%)
HLHS	22
MA	8
CAVC	6
DORV/CAVC	6
Complex	4
DORV	3
Single left ventricle physiology (n, %)	62 (53%)
TA	27
DILV	18
PAIVS	10
CAVC	3
Ebstein	2
PA + VSD	1
Complex	1
Indeterminate physiology (n, %)	6 (5.1%)
Criss-cross	5
Other	1
Dextrocardia (n, %)	10 (8.5%)
Right isomerism (n, %)	8 (6.8%)
Left isomerism (n, %)	7 (6%)
EC conduit (n, %)	100 (85.5%)
Lateral tunnel (n, %)	17 (14.5%)
Fenestration (n, %)	107 (91.5%)
Associated cardiac surgery (n, %)	28 (23.9%)
Perioperative complications	
Heart failure (n, %)	6 (5.1%)
Bleeding (n, %)	6 (5.1%)
Pleural effusion (n, %)	58 (49.6%)
Chylothorax (n, %)	8 (6.8%)
Infection (n, %)	18 (15.4%)
Kidney injury (n, %)	8 (6.8%)
Ascites (n, %)	3 (2.6%)
Arrhythmias (n, %)	10 (8.5%)
Percutaneous procedure during the follow-up period (n, %)	49 (41.9%)
Follow-up complications	
Arrhythmias (n, %)	37 (31.6%)
Pleural effusion (n, %)	4 (3.4%)
PB (n, %)	2 (1.7%)
Liver disease (n, %)	17 (14.5%)
PLE (n, %)	4 (3.4%)
Declivous oedema (n, %)	2 (1.7%)
Antithrombotic therapy	
Acetylsalicylic acid (n, %)	82 (70%)
Warfarin (n, %)	13 (11.1%)
Rivaroxaban (n, %)	6 (5%)

HLHS: hypoplastic left heart syndrome; MA: mitral atresia; CAVC: complete atrioventricular canal defect; DORV: double outlet right ventricle; TA: tricuspid atresia; DILV: double inlet left ventricle; PAIVS: pulmonary atresia with intact ventricular septum; PA + VSD: pulmonary atresia with ventricular septal defect; EC: extracardiac conduit; PB: plastic bronchitis; PLE: protein-losing enteropathy.

**Table 2:** Demographic and clinical characteristics of patients on pulmonary vasodilators.

Pt	Gender	CHD	Physiology	Age at Fontan (Months)	Type of Fontan	Which PVT	When	Why	Cath pre PAm–TPG–PVRi Anatomy	Stopped	Cath post PAm-TPG-PVRi	Duration (Years)
**1**	M	CAVC	SLV	42	EC	B	Perioperative	Low Sa02, PE, PA hypoplasia	6-5-1.11	Y	7-2-0.8	6
**2**	F	HLHS	SRV	55	EC	B	Months postoperative	PB	10-5-NR-circuit stenosis	Y	NR-2-0.6	5
**3**	M	MA	SRV	70	EC	B	Perinatal	TAPVR	-	N	9-4-2.9	-
**4**	F	TA	SLV	84	EC	B	One month postoperative	Persistent PE	6-3-2.09-Glenn stenosis and collaterals	N	-	-
**5**	F	Complex	SRV	65	EC	B S	Perioperative One year postoperative	Persistent PE Haemorrhagic alveolitis	8-2-0.8-PA hypoplasia -	Y N	- 11-5-1.4	Some months -
**6**	M	PAIVS	SLV	29	LT	B	Years postoperative	PB	8-5-1.6-circuit stenosis	N	8-3.5-0.83	-
**7**	F	TA	SLV	58	EC	B S	One year after birth Years preoperative	Low SaO2 Low SaO2	20-NR-NR-atrioseptostomy 13-5-3.5-hypoplasia PA	N N	- -	- -
**8**	M	MA	SRV	71	EC	S	Years postoperative	Haemoptysis	20-NR-NR-collaterals	N	-	-
**9**	M	HLHS	SRV	65	EC	B	Preintervention	High PVR	NR-NR-3.49	Y	12-NR-NR	4
**10**	F	CAVC	SRV	74	EC	S	One month after intervention (post PE)	Elevated circuit pressure	17-5-2.4	N	-	-
**11**	M	CAVC	SRV	55	EC	B	Preintervention	Elevated circuit pressure	15-NR-NR	Y	14-3.5-1.3	9
**12**	F	DORV	SRV	55	LT	B	Years postoperatively	PLE	NR-NR-0.9	N	13-5-1.9	-
**13**	M	CAVC	SRV	23	EC	B S	Years postoperatively Years postoperatively	Low SaO2 Low SaO2	12-6-3.33 13-4-NR	Y N	9-4-2-pulmonary fistulae -	1 -
**14**	M	DORV/CAVC	SRV	72	EC	S	Years postoperatively	Diastolic dysfunction	-	N	14-3-0.72	-
**15**	F	CRISS-CROSS	INDET	57	EC	S	Perioperatively	Elevated circuit pressure	23-NR-2.3-phasic flow	Y	-	0.25
**16**	M	EBSTEIN	SLV	72	EC	S	Years postoperatively	Elevated circuit pressure, poor exercise tolerance	15-7-2.3	N	-	-
**17**	M	DILV	SLV	64	EC	B	Years preoperatively	Slightly elevated circuit pressure	16-4-1.44-LPA stenosis	Y	14-NR-1.1	3
**18**	M	MA	SRV	64	EC	B	Months postoperatively	Chylothorax	18-NR-2.9-LPA and EC stenosis	N	-	-
**19**	M	HLHS	SRV	46	EC	B	Years postoperatively	Poor exercise tolerance	15-7-3.5	N	-	-
**20**	M	HLHS	SRV	86	EC	S	Months postoperatively	Pericardial effusion	15-5-2.5	Y	-	6

CHD: congenital heart disease; HLHS: hypoplastic left heart syndrome; MA: mitral atresia; CAVC: complete atrioventricular connection; DORV: double outlet right ventricle; TA: tricuspid atresia; DILV: double inlet left ventricle; PAIVS: pulmonary atresia with intact ventricular septum; TAPVR: total anomalous pulmonary venous return; PB: plastic bronchitis; PLE: protein-losing enteropathy; SLV: single left ventricle; SRV: single right ventricle; EC: extracardiac conduit; LT: lateral tunnel; B: bosentan; S: sildenafil; PAm: mean pulmonary arterial pressure; TPG: trans pulmonary gradient; PVRi: indexed pulmonary vascular resistance; Y: yes; N: no; NR: not reported; PE: pleural effusion; PA: pulmonary arteries, LPA: left pulmonary artery; PVT: pulmonary vasodilator therapy.

**Table 3:** Demographic and anatomical characteristics and perioperative and follow-up data: pulmonary vasodilators vs. no vasodilators.

	Treated	Not Treated	*p*-Values
Female (n, %)	7 (35%)	36 (37.1%)	1
Age at intervention, months (median, IQR)	64 (55–71.25)	46 (36–61)	**0.05**
Single right ventricle physiology (n, %)	13 (65%)	36 (37%)	**0.03**
HLHS	4 (20%)	18 (18.5%)	1
MA	3 (15%)	5 (5.2%)	0.14
CAVC	3 (15%)	3 (3%)	0.06
DORV/CAVC	1 (5%)	5 (5.2%)	1
Complex	1 (5%)	3 (3%)	0.5
DORV	1 (5%)	2 (2.1%)	0.4
Single left ventricle physiology (n, %)	6 (30%)	56 (47.9%)	**0.03**
TA	2 (10%)	25 (25.8%)	0.15
DILV	1 (5%)	17 (17.5%)	0.3
PAIVS	1 (5%)	9 (9.3%)	1
CAVC	1 (5%)	2 (2.1%)	0.4
Ebstein	1 (5%)	1 (1%)	0.3
PA+VSD	0	1 (1%)	1
Complex	0	1 (1%)	1
Indeterminate physiology (n, %)	1 (5%)	5 (5.2%)	1
Criss-cross	1 (5%)	4 (4%)	1
Other	0	1 (1%)	1
Dextrocardia (n, %)	3 (15%)	7 (7.2%)	0.37
Right isomerism (n, %)	2 (10%)	6 (6.2%)	0.6
Left isomerism (n, %)	1 (5%)	6 (6.2%)	1
EC conduit (n, %)	18 (90%)	82 (84.5%)	0.7
Lateral tunnel (n, %)	2 (10%)	15 (15.5%)	0.7
Fenestration (n, %)	19 (95%)	88 (90.7%)	1
Associated cardiac surgery (n, %)	7 (35%)	21 (21.6%)	0.25
Perioperative complications			
Heart failure (n, %)	0	6 (6.2%)	0.6
Bleeding (n, %)	2 (10%)	4 (4%)	0.6
Pleural effusion (n, %)	12 (60%)	46 (47%)	0.3
Chylothorax (n, %)	3 (15%)	5 (5.2%)	0.1
Infection (n, %)	6 (30%)	12 (12.4%)	0.08
Kidney injury (n, %)	3 (15%)	5 (5.2%)	0.1
Ascites (n, %)	1 (5%)	2 (2.1%)	0.4
Arrhythmias (n, %)	4 (20%)	6 (6.2%)	0.06
Follow-up duration, months (median, IQR)	96 (78.5–138)	158 (96–215)	**0.05**
Percutaneous procedure during the follow-up period (n, %)	17 (85%)	32 (33%)	**0.0001**
Follow-up complications			
Arrhythmias (n, %)	10 (50%)	27 (27.8%)	0.06
Pleural effusion (n, %)	1 (5%)	3 (3%)	0.5
PB (n, %)	2 (10%)	0	**0.03**
Liver disease (n, %)	6 (30%)	11 (11.3%)	0.07
PLE (n, %)	2 (10%)	2 (2.1%)	0.13
Declivous oedema (n, %)	2 (10%)	0	**0.03**
Antithrombotic therapy			
Acetylsalicylic acid (n, %)	11 (55%)	71 (73%)	0.11
Warfarin (n, %)	7 (35%)	6 (6.2%)	**0.001**
Rivaroxaban (n, %)	2 (10%)	4 (4%)	0.3

HLHS: hypoplastic left heart syndrome; MA: mitral atresia; CAVC: complete atrioventricular canal defect; DORV: double outlet right ventricle; TA: tricuspid atresia; DILV: double inlet left ventricle; PAIVS: pulmonary atresia with intact ventricular septum; PA + VSD: pulmonary atresia with ventricular septal defect, PB: plastic bronchitis; PLE: protein-losing enteropathy. Bold font to highlight statistically significant *p*-values.

## Data Availability

The raw data supporting the conclusions of this article will be made available by the authors upon request. The data are not publicly available due to privacy reason.
